# Fibulin-4 is associated with tumor progression and a poor prognosis in ovarian carcinomas

**DOI:** 10.1186/s12885-015-1100-9

**Published:** 2015-03-04

**Authors:** Jie Chen, Zhao Liu, Shuang Fang, Rui Fang, Xi Liu, Yueran Zhao, XiangXin Li, Lei Huang, Jie Zhang

**Affiliations:** 1 Department of Maternal and Child Health Care, School of Public Health, Shandong University, Jinan, 250012 China; 2 Hepatobiliary and Pancreatic Surgery, Jinan Central Hospital affiliated to Shandong University, Jinan, 250013 China; 3 Biochemistry & Molecular Biology, Georgetown University, Georgetown, Washington D.C 20057 USA; 4 Grade 2011, Clinical Medicine, School of Medicine, Shandong University, Jinan, 250012 China; 5 Central Laboratory, Shandong Provincial Hospital affiliated to Shandong University, Jinan, 250021 China; 6 Department of Haematology, QiLu Hospital of Shandong University, Jinan, 250012 China; 7 Department of Pediatrics, Shandong Provincial Hospital affiliated to Shandong University, Jinan, 250021 China

**Keywords:** Fibulin-4, Ovarian carcinoma, Angiogenesis, Prognosis

## Abstract

**Background:**

Fibulin-4, a member of the fibulin family of extracellular glycoproteins, is implicated in the progressions of some cancers. However, no information has been available to date regarding the function of fibulin-4 in ovarian carcinoma progression.

**Methods:**

In this study, fibulin-4 mRNA and protein expression in normal ovarian tissue, ovarian tumor, high invasive subclones and low invasive subclones were evaluated by immunohistochemistry and real time reverse transcriptase-polymerase chain reaction (RT-PCR). The serum levels of fibulin-4, cancer antigen 125 (CA-125) and cerbohydrate antigen 199 (CA19-9) in patients with ovarian tumor were measured by enzyme-linked immunosorbent assay and electrochemiluminescent immunoassay. To assess the angiogenic properties of fibulin-4, vascular endothelial growth factor (VEGF) expression and tumor microvessel density were analyzed in ovarian carcinoma by immunohistochemistry.

**Results:**

Fibulin-4 expression was upregulated in ovarian carcinoma, and positively correlated with MVD and VEGF expression. Fibulin-4 overexpression was significantly associated with advanced stage, low differentiation, lymph node metastasis and poor prognosis in patients with ovarian cancer. The serum levels of fibulin-4, CA-125 and CA19-9 in patients with ovarian carcinoma were much higher than those with benign ovarian tumors and normal controls. Compared to CA-125 and CA19-9, fibulin-4 had better diagnostic sensitivity and specificity.

**Conclusions:**

Fibulin-4 is a novel gene that is found overexpressed in ovarian cancer and associated with poor prognostic clinicopathologic features. This study shows that fibulin-4 may serve as a new prognostic factor and as a potential therapeutic target for patients with ovarian cancer in the future.

## Background

Ovarian cancer is one of the most aggressive and heterogeneous cancer types in women and one of the leading causes of gynaecological deaths [1,2]. Its high mortality is attributable to the fact that the majority of ovarian cancer patients are diagnosed at advanced stages when conventional therapy is less effective [3]. Although substantial advances have been made in ovarian cancer research, the overall 5-year survival rate is still less than 30% [4]. Tumor recurrence and metastasis are considered the major reasons for poor clinical outcome and cancer deaths [5]. Therefore, studying the mechanism of tumor invasion and metastasis will provide further insights into the development and progression of ovarian cancer. In recent years, many biomarkers have been investigated which are involved in the progression of ovarian cancer [6]. But few studies have been done to assess the functions of fibulin-4 in ovarian cancer development.

Fibulin-4, also known as endothelial growth factor (EGF)-containing fibulin-like extracellular matrix protein 2 (EFEMP2), mutant p53 binding protein 1 (MBP1), or UPH1, is a 443 amino acid secreted protein that contains six EGF-like calcium-binding domains and belongs to the fibulin family [7]. Fibulins have been shown to modulate cell morphology, growth, adhesion and motility, and are closely associated with the development of a wide variety of carcinomas [8]. As tumor suppressor genes, fibulin-2 [9,10] and fibulin-5 [11-13] were widely considered to be associated with the suppression of tumor growth, invasion, and angiogenesis. The research findings on the role of fibulin-1 and fibulin-3 in different tumor tissues have been controversial. Few researchers reported oncogenic activities [14-20], whereas others have reported tumor-suppressive activities [21-28]. This discrepancy may be attributable to the influence of the tumor microenvironment on tumor-associated genes in promoting angiogenesis and metastasis [29].

Fibulin-4 is essential for connective tissue development and elastic fiber formation and may also play an important role in vascular patterning and collagen biosynthesis [30]. Fibulin-4 plays a role in many clinical conditions such as cutis laxa [31], aortic aneurysms [32], osteoarthritis [33], and cancer [34,23]. In the study on colon tumors [34], Gallagher et al. found that the fibulin-4 gene was localized on chromosome 11q13; translocations, amplifications, and other rearrangements in this region are associated with a variety of human cancers [35,36]. Reverse transcriptase (RT)-polymerase chain reaction (PCR) of RNA from paired human colon tumors and adjacent normal tissue showed that tumors had a 2–7 fold increase in the level of fibulin-4 mRNA expression [34]. However, in prostate cancer [23], fibulin-4 is significantly downregulated and is weakly expressed in carcinoma cell lines compared to normal prostate epithelial cells. Against this background of controversies in the research addressing the role of fibulin-4, more studies are needed to elucidate the relationship between fibulin-4 and cancer. To our knowledge, the role of fibulin-4 in cervical cancer remains unexplored.

The purpose of this study was to assess whether fibulin-4 expression was associated with the progression of ovarian cancer, and further to investigate the relationship between fibulin-4 and angiogenesis.

## Methods

### Cell lines

Highly invasive subclones (S1, A1) and low invasive subclones (S21, A19) were derived from the SKOV3 and 3AO human ovarian cancer cell lines, using the limited dilution method. Next, the cell electrophoretic mobility (EPM) of each clone was measured to study the charge-related properties using microcapillary electrophoresis chips according to Omasu’s methods [37]. Finally, the MTT assay, soft agar colony formation assay, matrigel invasion assay, and cell migration assay were performed and tumor xenografts were generated in nude mice to confirm that high invasive subclones and low invasive subclones had high and low metastatic potential, respectively [38]. Cells were cultured in RPMI-1640 supplemented with 10% fetal bovine serum (FBS) and antibiotics (Gibco BRL, Rockville, MD).

### Tissue specimens

A total of 260 human ovarian tissue specimens obtained with written informed consent from patients were used for this study. Two hundred and twenty (220) epithelial ovarian tumors were enrolled from the Department of Gynecology and Obstetrics, Shandong Provincial Hospital between 2005 and 2011. There were 60 benign ovarian tumors that contain 25 serous cystadenoma, 22 mucinous cystadenoma and 13 endometrioid tumor (age range, 20–45 years; mean [SD], 35 [6] years) and 160 epithelial ovarian carcinomas that contain 58 serous cystadenocarcinoma, 56 mucinous cystadenocarcinoma and 46 endometrioid carcinoma (age range, 28–65 years; mean [SD], 42 [8] years). All ovarian cancer patients were clinically staged according to the International Federation of Gynecology and Obstetrics (FIGO) staging system (FIGO stage I, 36 cases; FIGO stage II, 38 cases; and FIGO stage III, 46 cases; and FIGO stage IV, 40 cases). None of the ovarian cancer patients received preoperative radiation or chemotherapy. All patients were treated consecutively and were followed up regularly; 5 patients were lost to follow-up and 20 patients died during the study period. Follow-up duration was between 1 to 7 years by the end of 2012. Forty normal ovary tissue specimens (age range, 25–65 years; mean [SD], 45 [7] years) were obtained from the Department of Gynecology and Obstetrics, Shandong Provincial Hospital. The study was approved by the Institutional Medical Ethics Committee of Shandong University.

### Blood samples

Blood samples were accordingly obtained with the written informed consent from the same 220 ovarian tumor patients that contain 60 benign ovarian tumors and 160 epithelial ovarian carcinomas at the Department of Gynecology and Obstetrics, Shandong Provincial Hospital between 2005 and 2011. None of the ovarian cancer patients received preoperative radiation or chemotherapy. Blood samples were collected before the initiation of treatment and centrifuged at 1500 g for 10 minutes. Aliquots of the separated plasma were stored at −80°C for future analysis. Forty control blood samples were obtained with the written informed consent from age-matched examinees undergoing health examinations at Shandong Provincial Hospital. Control subjects had no history of disease and no abnormalities on laboratory examinations. The study was approved by the Institutional Medical Ethics Committee of Shandong University.

### Enzyme-linked immunosorbent assay

Levels of fibulin-4 in serum samples were measured using sandwich enzyme-linked immunosorbent assay (ELISA) with human fibulin-4 ELISA assay kits (Immuno-Biological Laboratories, Japan). Serum was diluted with Enzyme ImmunoAssay (EIA) buffer (1% BSA, 0.05% Tween 20 in phosphate buffer) and incubated for 2 hour at 37°C. After 4 washes with EIA buffer, horse radish peroxidase-conjugated antibodies were added and incubated for 30 minutes at 4°C. After washed 4 times, 100 μl of tetramethyl benzidine solution was added and incubated for 30 minutes at room temperature. The reaction was stopped with 100 μl of 1 N sulfuric acid and measured using the ELISA reader at 450 nm.

### Quantitative analysis of CA-125 and CA19-9

Serum CA-125 and CA19-9 were detected using the electrochemiluminescent immunoassay (ECLIA) method. The ECLIA kits were provided by Roche Diagnostics (Mannheim, Germany) and Roche E170 electrochemiluminescent analyzer was used as the instrument with 20 μl per serum sample.

### Immunohistochemistry (IHC)

According to the standard streptavidin-biotin-peroxidase complex procedures, immunohistochemistry (IHC) was performed on formalin-fixed, paraffin-embedded sections (5 μm thick) and cell slides were fixed in 4% paraformaldehyde. Briefly, after dewaxing, rehydration, and antigen retrieval, the sections were incubated with rabbit anti-human fibulin-4 monoclonal antibody (ab125073, Abcam) with working dilutions of 1: 200 at 4°C overnight. Human breast cancer paraffin-embedded sections (fibulin-4 positive) were used as positive controls. A negative control was obtained by replacing the primary antibody with normal rabbit immunoglobulin (IgG). Positive expression of fibulin-4 protein was defined as the presence of brown granules in the cytoplasm.

### Immunohistochemistry (IHC) analysis

A semiquantitative scoring system derived from the method by Soumaoro [39] for both the intensity of staining and the percentage of positive cells was used to evaluate fibulin-4 expression. The intensity of fibulin-4 positive staining was scored from 0 to 3 (negative = 0, weak = 1, moderate = 2, or strong = 3) and the percentage of positively stained cells was scored as 0 (0%), 1 (1–25%), 2 (26–50%), 3 (51–75%), and 4 (76–100%). The sum of the intensity and percentage scores was used as the final staining scores (0 to 7). The sum-indexes (−), (+), (++), and (+++) indicated final staining scores of 0, 1–3, 4–5, and 6–7, respectively. For statistical analysis, sum-indexes (−) and (+) were defined as low fibulin-4 expression, while sum-indexes (++) and (+++) were defined as high fibulin-4 expression. Each section was independently scored by three pathologists. To assess reproducibility, we invited three other pathologists to score all sections independently. The interobserver reliability and intraobserver reproducibility of IHC experiments were evaluated by kappa statistic evaluation.

### Microvessel assessment

Microvessel density (MVD) was assessed according to CD31 immunohistochemical staining of tumor vessels. Any immune-positive single endothelial cell or endothelial cell clusters and microvessels in the tumor were considered to be individual vessels and were counted, as described by Weidner et al. [40]. Peritumoral vascularity, vascularity in areas of necrosis and vessels with a thick muscle wall or in a diameter larger than eight erythrocytes, was not counted. The sections were scanned at low power (100×) to select the most vascularized (hot-spots) areas. The microvessels in the hot-spots were then counted, and an average count in three hot spots was calculated as the MVD. All counts were made independently by three observers who were blinded to the corresponding clinicopathologic data.

### Quantitative real-time-polymerase chain reaction

Total RNA was extracted using Trizol reagent (Invitrogen) and reverse transcribed. Quantitative real-time PCR analysis was performed using ABI PRISM 7500 Real-Time PCR System (Applied Biosystems). Each well (20 μl reaction volume) contained 10 μl Power SYBR Green PCR master mix (Applied Biosystems), 1 μl of each primer (5 μmol/l) and 1 μl template. The following primers were used:

- fibulin-4 5′- GCTGCTACTGTTGCTCTTGGG -3′
- 5′- GGGATGGTCAGACACTCGTTG -3′
- β-actin 5′-CCACGAAACTACCTTCAACTCCA-3′
- 5′-GTGATCTCCTTCTGCATCCTGTC-3′

### Western blot

Cells were lysed by using RIPA buffer containing 1 mM PMSF. Fifty microgram of protein per lane was resolved by SDS-PAGE and transferred to PVDF membrane and blocked with 5% BSA. After incubating with primary antibody to goat human fibulin-4 and VEGF overnight at 4°C and horseradish peroxidase-conjugated anti-goat IgG as secondary antibody for 1 hour at room temperature, blots were developed using ECL method. Band intensity was analyzed using Gel-Pro Analyzer Software (Media Cybernetics, Inc., Bethesda, MD).

### Statistical analysis

IHC data were analyzed using the chi-square test. Measurement data were expressed as the mean ± SE. The interobserver reliability and intraobserver reproducibility of IHC experiments were evaluated using kappa statistic evaluation. The strength of agreement was interpreted as follows: excellent (kappa ≥ 0.80), good (0.60–0.79), moderate (0.40–0.59), poor (0.20–0.39), and very poor (<0.20) [41]. For comparison of means between two groups, a two-tailed t-test was used and for comparison of means among three groups, one-way ANOVA was used. Survival curves were calculated using the Kaplan-Meier method and analyzed using the log-rank test. Correlations of fibulin-4 expression with VEGF expression and MVD were analyzed using the Pearson correlation test. Multivariate Cox proportional hazard models were used to define the potential prognostic significance of individual parameters. Receiver-operating characteristic (ROC) curve was performed and the area under the curve (AUC) was calculated separately to test the sensitivity and specificity of all three biomarkers. The value of AUC is between 0.5 and 1, and the diagnostic accuracy was interpreted as follows: good (AUC ≥ 0.90), moderate (0.70–0.89) and poor (0.50–0.69). Statistical analysis was performed using SPSS software version 13.0. Two-sided p values of <0.05 were considered statistically significant.

## Results

### Fibulin-4 expression in human ovarian tissues

**Figure 1:**
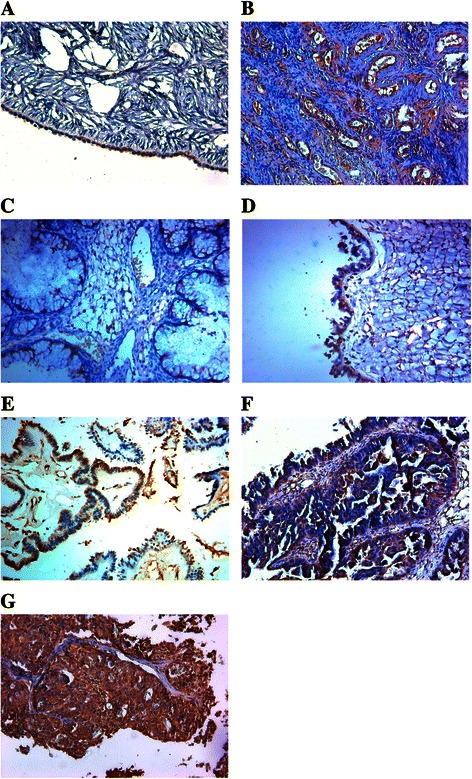
**Expressions of fibulin**-**4 in human ovarian tissues.(A)** The epithelial cells of normal human ovarian, **(B)** the stroma of normal human ovarian, **(C, D)** Benign ovarian tumor, **(E)** High differentiation of ovarian carcinoma, **(F)** Medium differentiation of ovarian carcinoma, **(G)** Low differentiation of ovarian carcinoma. (Magnification × 200).

**Figure 2:**
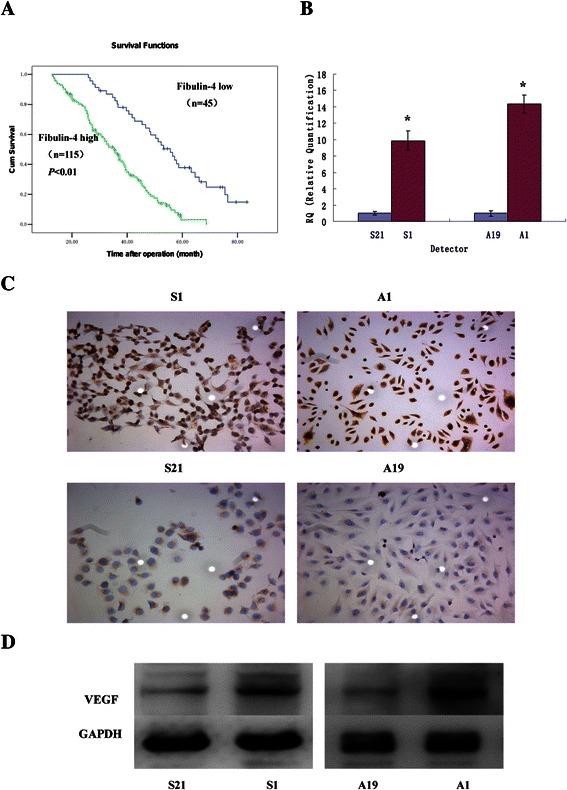
**Kaplan-Meier analysis and VEGF expressions in highly invasive subclones and low invasive subclones.(A)** 160 patients with invasive cancer were included in the cohort. Patients with high EFEMP1 expression (green line, n = 115) had a much worse prognosis than those with low EFEMP1 expression (blue line, n = 45). **(B)** Fibulin-4 mRNA expressions of highly invasive subclones S1 and A1 and low invasive subclones S21 and A19 as measured by q-RT-PCR. **(C)** Fibulin-4 protein expressions of highly invasive subclones and low invasive subclones as measured by ICC staining (Magnification × 200). **(D)** Fibulin-4 protein expressions of highly invasive subclones and low invasive subclones as measured by Western blot. *P < 0.05 versus control.

As shown in Figure 1, in normal human ovarian surface epithelial cells, fibulin-4 protein expression was very low (Figure 1A), and in the ovarian stroma, fibulin-4 protein expression was mainly focused around the vasculatures (Figure 1B). However in most ovarian carcinomas, fibulin-4 immunoreactivity was high, and high fibulin-4 protein expression was found in the cytoplasm of ovarian cancer cells (Figure 1E,F,G). Moreover, high fibulin-4 protein expression was associated with low differentiation, advanced stage and positive lymph node status of ovarian carcinomas (Table 1). The interobserver reliability coefficients were 0.84 and 0.87 for the first and second assessments, with an intraobserver reproducibility coefficient of 0.86. The interobserver reliability and intraobserver reproducibility of IHC experiments were excellent. Similar results were also shown in the real time RT-PCR experiment, fibulin-4 mRNA expression was also very low in normal ovarian tissues and benign ovarian tumors, and significantly high fibulin-4 expression was seen in ovarian carcinoma. Moreover, high fibulin-4 mRNA expression was also associated with low differentiation, advanced stage and positive lymph node status of ovarian carcinomas (Table 2). To evaluate the prognostic value of fibulin-4 in ovarian cancer, we performed survival analysis using Kaplan-Meier analysis. The result showed that patients with high fibulin-4 expression had a much worse prognosis than those with low fibulin-4 expression (log rank, P < 0.01; Figure 2A). In multivariate analysis, considering all histological and molecular features together, the important prognostic factors were fibulin-4 expression (P = 0.000; hazard ratio 2.129), lymph node metastasis (P = 0.001; hazard ratio 1.017), and tumor stage (P = 0.005; hazard ratio 1.984) (Table 3).

**Table 1:** **Protein expression of fibulin**-**4 in human ovarian tissues**

	N	Fibulin-4 low (−/+)		Fibulin-4 high (++/+++)		*X* ^*2*^	*P*
		n	%	n	%		
Normal	40	37	92.5%	3	7.5%	65.455	<0.01
Benign	60	40	66.7%	20	33.3%		
Pathology type						0.141	0.932
*Serous cystadenoma*	25	16	64%	9	36%		
*Mucinous cystadenoma*	22	15	68.2%	7	31.8%		
*Endometrioid tumor*	13	9	69.2%	4	30.8%		
Carcinoma	160	45	28.1%	115	71.9%		
Pathology type						0.736	0.692
*Serous cystadenocarcinoma*	58	16	27.6%	42	72.4%		
*Mucinous cystadenocarcinoma*	56	14	25%	42	75%		
*Endometrioid carcinoma*	46	15	32.6%	31	67.4%		
Cell differentiation						32.987	<0.01
*High and Medium*	88	41	46.6%	47	53.4%		
*Low*	72	4	5.6%	68	94.4%		
Tumor stage						21.629	<0.01
*Low stage*	74	34	45.9%	40	54.1%		
*High stage*	86	11	12.8%	75	87.2%		
Nodal status						35.752	<0.01
*Positive*	83	8	9.6%	75	90.4%		
*Negative*	77	41	53.2%	36	46.8%

**Table 2:** **mRNA expression of fibulin**-**4 in human ovarian tissues**

	N	Fibulin-4 mRNA	*P*
Control	40	0.0089 ± 0.0047	
Benign	60	0.0092 ± 0.0054	>0.05
Pathology type			>0.05
*Serous cystadenoma*	25	0.0091 ± 0.0058	
*Mucinous cystadenoma*	22	0.0098 ± 0.0067	
*Endometrioid tumor*	13	0.0084 ± 0.0035	
Carcinoma	16	0.0947 ± 0.0083	<0.05
Pathology type	0		>0.05
*Serous cystadenocarcinoma*	58	0.0872 ± 0.0097	
*Mucinous cystadenocarcinoma*	56	0.0913 ± 0.0108	
*Endometrioid carcinoma*	46	0.0894 ± 0.0087	
Cell differentiation			<0.05
*High and Medium*	88	0.0257 ± 0.0084	
*Low*	72	0.0968 ± 0.0113	
Tumor stage			<0.05
*Low stage*	74	0.0284 ± 0.0075	
*High stage*	86	0.0895 ± 0.0118	
Nodal status			<0.05
*Positive*	83	0.0983 ± 0.0094	
*Negative*	77	0.0309 ± 0.0081

**Table 3:** **Predictive factors of survival by multivariate analysis** (**Cox proportional hazards model**)

Prognostic factors	Hazard ratio(95%CI)	P
Fibulin-4	3.573(2.033, 6.282)	0.000
Pathology type	1.263(0.593, 2.689)	0.545
Cell differentiation	1.095(0.986, 1.216)	0.089
Tumor stage	1.984(1.236, 3.185)	0.005
Lymph node metastasis	1.017(1.007, 1.027)	0.001
Tumor size	1.012(0.999, 1.026)	0.065
Age	1.263(0.593, 2.689)	0.545

### Different expression of fibulin-4 and VEGF in the highly invasive subclone and low invasive subclone

**Figure 3:**
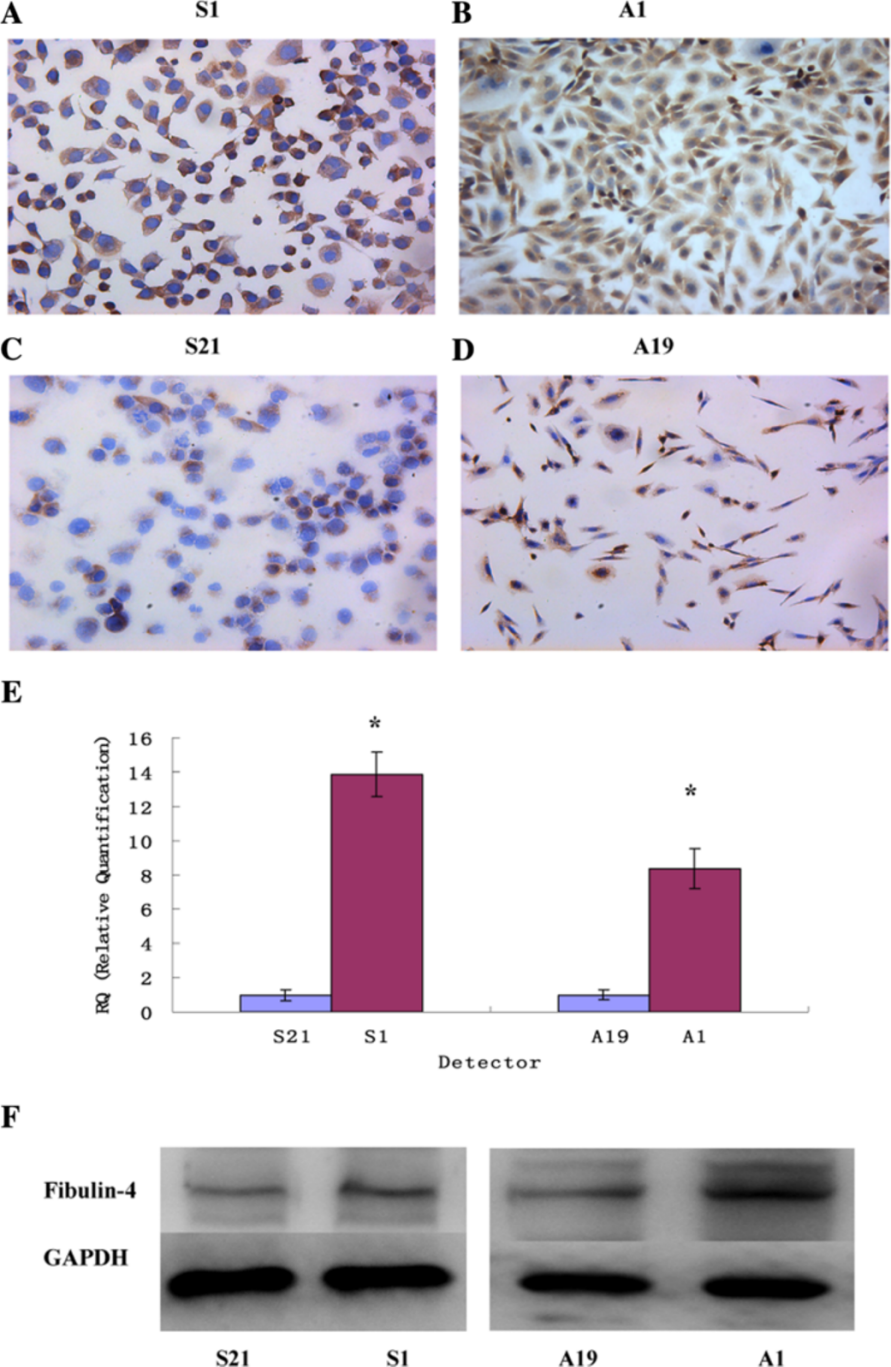
**Fibulin-4 expressions in highly invasive subclones and low invasive subclones.** (ABCD) Fibulin-4 protein expressions of highly invasive subclones S1 **(A)** and A1 **(B)** and low invasive subclones S21 **(C)** and A19 **(D)** as measured by ICC staining (Magnification × 200). **(E)** Fibulin-4 mRNA expressions of highly invasive subclones and low invasive subclones as measured by q-RT-PCR. **(F)** Fibulin-4 protein expressions of highly invasive subclones and low invasive subclones as measured by Western blot. *P < 0.05 versus control.

The highly invasive subclone (S1 and A1) and the low invasive subclone (S21 and A19) were derived from the SKOV3 and 3AO human ovarian cancer cell lines, using the limited dilution method. Since the cell lines have had similar genetic backgrounds, they are suitable for comparative analysis. As shown in Figure 2B,C,D and Figure 3, the protein and mRNA expressions of fibulin-4 and VEGF were much higher in highly invasive subclones (S1 and A1), compared to the low invasive subclones (S21 and A19).

### Serum levels of fibulin-4, CA-125 and CA19-9 in human ovarian tumor patients and healthy controls

**Figure 4:**
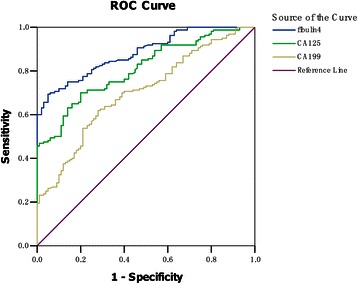
**Receiver operator characteristic(ROC)curves of fibulin-4,CA**-**125 and CA19**-**9 in patients with ovarian cancer.** The area under the curve (AUC) of fibulin-4, CA-125 and CA19-9 were 0.883, 0.808 and 0.701, suggesting their clinical usefulness for diagnosing ovarian carcinoma was moderate.

As shown in Table 4, the serum levels of fibulin-4, CA-125 and CA19-9 in patients with ovarian carcinoma was much higher than that with benign ovarian tumor and healthy controls (*P* < 0.05). No significant difference was found between healthy control and benign ovarian tumor (*P* >0.05). Moreover, high serum levels of fibulin-4, CA-125 and CA19-9 were associated with low differentiation, advanced stage and positive lymph node status of ovarian carcinomas (*P* < 0.05). There were no significant differences in the serum levels of fibulin-4 among different pathology types of ovarian carcinoma (P >0.05). However, the serum level of CA-125 was increased in serous cystadenocarcinoma and CA19-9 was increased in mucinous cystadenocarcinoma (P < 0.05). The serum levels of fibulin-4, CA-125 and CA19-9 were evaluated by ROC analysis (Figure 4). The AUC of fibulin-4, CA-125 and CA19-9 were 0.883, 0.808 and 0.701, suggesting that clinical usefulness of the three biomarkers for diagnosing ovarian carcinoma was moderate. The Youden index [42] identified the cut-off level of fibulin-4 was 45.79 ng/ml, with a sensitivity of 75.0% and a specificity of 84.0%. Table 5 shows the comparisons of sensitivity, specificity, positive predictive value, negative predictive value, positive likelihood ratio and negative likelihood ratio among the three markers. In combined measurements, when 2 markers were both determined in diagnosis of ovarian cancer, combination of fibulin-4 and CA-125 was superior to other two combinations. When combined fibulin-4, CA-125 and CA19-9, the diagnostic specificity, positive predictive value and positive likelihood ratio were all significantly increased.

**Table 4:** **Serum levels of fibulin**-**4**, **CA**-**125 and CA19**-**9 in patients with ovarian tumor**

	N	Fibulin-4(ng/ml)	P	CA-125(U/ml)	P	CA19-9(U/ml)	P
Control	40	29.54 ± 16.17		33.32 ± 24.55		35.67 ± 15.59	
Benign	60	38.15 ± 18.43	>0.05	33.16 ± 16.23	>0.05	34.89 ± 17.26	>0.05
Pathology type			>0.05		>0.05		>0.05
*Serous cystadenoma*	25	37.26 ± 12.54		30.51 ± 10.83		36.85 ± 16.27	
*Mucinous cystadenoma*	22	36.75 ± 14.32		35.24 ± 12.69		33.92 ± 14.78	
*Endometrioid tumor*	13	39.26 ± 19.73		32.76 ± 15.93		31.61 ± 13.41	
Carcinoma	160	267.06 ± 238.71	<0.05	231.60 ± 205.47	<0.05	158.21 ± 124.59	<0.05
Pathology type			>0.05		<0.05		<0.05
*Serous cystadenocarcinoma*	58	273.65 ± 215.87		366.22 ± 216.54		97.32 ± 31.13	
*Mucinous cystadenocarcinoma*	56	259.68 ± 211.69		144.38 ± 95.53		275.63 ± 107.69	
*Endometrioid carcinoma*	46	265.72 ± 207.94		138.46 ± 84.95		89.86 ± 49.37	
Cell differentiation			<0.05		<0.05		<0.05
*High and Medium*	88	104.58 ± 83.86		123.86 ± 90.22		87.45 ± 55.36	
*Low*	72	363.29 ± 239.63		378.29 ± 197.34		255.64 ± 158.12	
Tumor stage			<0.05		<0.05		<0.05
*Low stage*	74	113.31 ± 96.05		128.73 ± 85.59		73.59 ± 40.64	
*High stage*	86	364.37 ± 243.92		388.61 ± 216.33		247.38 ± 146.55	
Nodal status			<0.05		<0.05		<0.05
*Positive*	83	353.94 ± 214.37		376.48 ± 225.64		268.93 ± 117.32	
*Negative*	77	101.55 ± 86.81		131.45 ± 99.56		92.78 ± 61.19

**Table 5:** **Comparison of the diagnostic performance of serum fibulin**-**4**, **CA**-**125**, **CA19**-**9**, **fibulin**-**4** + **CA**-**125**, **fibulin**-**4** + **CA19**-**9**, **CA**-**125** + **CA19**-**9 and fibulin**-**4** + **CA**-**125** + **CA19**-**9**

Marker	Sensitivity (%)	Specificity (%)	Positive predictive value (%)	Negative predictive value (%)	Positive likelihood ratio	Negative likelihood ratio
fibulin-4	75.0	84.0	88.2	67.7	4.69	0.30
CA-125	70.6	79.0	84.3	62.7	3.36	0.37
CA19-9	61.3	70.0	76.6	53.0	2.04	0.55
fibulin-4 + CA-125	68.8	92.0	93.2	64.8	8.59	0.34
fibulin-4 + CA19-9	60.6	90.0	90.6	58.8	6.06	0.44
CA-125 + CA19-9	56.3	88.0	88.2	55.7	4.69	0.50
fibulin-4 + CA-125 + CA19-9	52.5	98.0	97.7	56.3	26.25	0.48

### Relationships of fibulin-4 with VEGF expression and MVD

**Figure 5:**
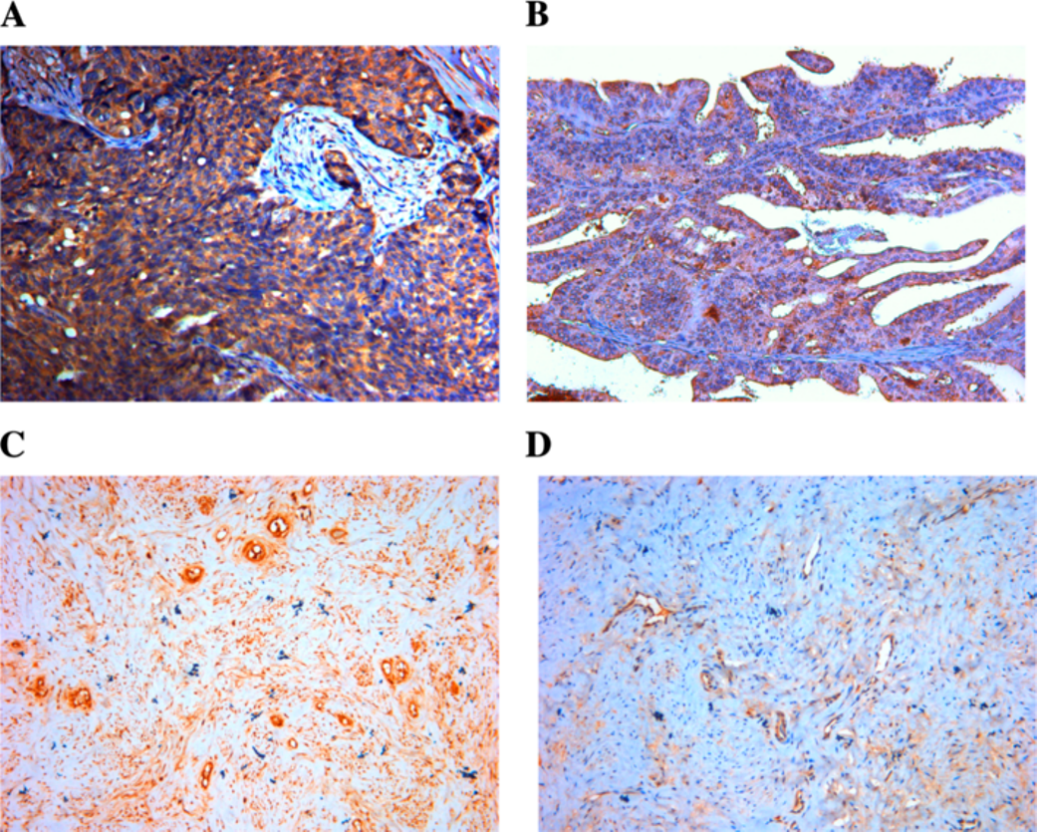
**Immunohistochemical staining of VEGF and CD34 for MVD.** Immunohistochemical staining of VEGF in low differentiation of ovarian carcinoma **(A)**, and high differentiation of ovarian carcinoma **(B)**. (Magnification × 200). Immunohistochemical staining of CD34 for MVD in low differentiation of ovarian carcinoma **(C)**, and high differentiation of ovarian carcinoma **(D)**. (Magnification × 200).

**Figure 6:**
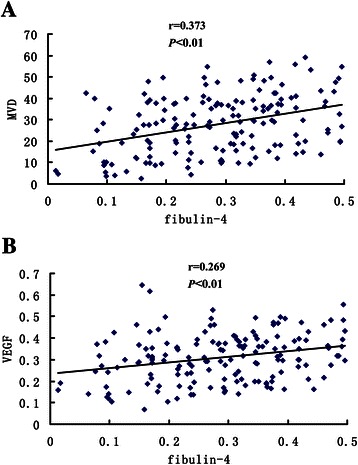
**Pearson correlations analysis of fibulin-4 expression with MVD and VEGF.** The expression of fibulin-4 positively correlated with MVD **(A)** and VEGF **(B)**.

Figure 5 shows the representative immunohistochemical staining images of VEGF and CD34. The immunohistochemical expressions of VEGF and fibulin-4 were evaluated with software Imag Pro Plus 6.0 to detect the photodensity. In brief, five positive fields in a section were selected at random and then read using Imag Pro Plus 6.0, finally the average densities were calculated. Pearson correlation tests of MVD (Figure 6A, *P* < 0.01) and VEGF expression (Figure 6B, *P* < 0.01) versus fibulin-4 revealed strong positive correlations.

## Discussion

In the present study, we have demonstrated for the first time that the expression of fibulin-4 is associated with poor prognostic clinicopathologic features, neovascularization, and poor outcomes in human ovarian carcinomas.

Our immunohistochemical studies showed an up-regulation of fibulin-4 expression in ovarian carcinoma tissues, compared with normal ovarian tissues and benign ovarian tumors. Real time PCR results confirmed that mRNA expression of fibulin-4 was also up-regulated in ovarian carcinoma tissues. Moreover, high fibulin-4 expression was associated with low differentiation, high stage and positive lymph node status in ovarian carcinomas. Similar results have been reported in earlier studies on colon cancer; dysregulated expression of the fibulin-4 gene was shown to be associated with human colon tumourigenesis [34]. However, contrasting results have also been reported for prostate cancer. By microarray analysis, the fibulin-4 genes were significantly down-regulated in prostate cancer and this result was corroborated by quantitative RT-PCR [35]. In our study, fibulin-4 was overexpressed in ovarian carcinomas and was shown to play an important role in tumor development. As is the case for other fibulins, there are controversies in research on fibulin-4; these discrepancies may be attributable to the fact that the tumor microenvironment influences the functions of tumor-associated genes [29].

Angiogenesis is the process of formation of new microvessels from preexisting vasculature. Once the tumor volume exceeds a few millimeters in diameter, hypoxia and nutrient deprivation trigger tumor cells to exploit their microenvironment by releasing cytokines and growth factors, which then activate normal, quiescent cells around them and initiate a cascade of events resulting in tumor progression. For example, tumor cell–derived VEGF stimulates the sprouting and proliferation of endothelial cells. VEGF is considered the most potent candidate for angiogenesis induction during tumor growth [43]. Since angiogenesis is essential for tumor growth and metastasis, controlling tumor-associated angiogenesis is a promising strategy for inhibiting cancer progression. In our study, we sought to determine whether fibulin-4 is associated with angiogenesis. So the Pearson correlation coefficient was calculated to assess the correlation of fibulin-4 with MVD and VEGF expression. We found that fibulin-4 expression was positively correlated with MVD and VEGF expression, and the expressions of fibulin-4 and VEGF were both much higher in highly invasive subclones than in low invasive subclones, which indicated that fibulin-4 might promote angiogenesis. No earlier studies on fibulin-4 had reported an association with tumor angiogenesis, although its highly homologous proteins, fibulin-3 and fibulin-5 were found to be associated with tumor angiogenesis. For example, exogenous and endogenous fibulin-5 was shown to be anti-angiogenic [44]. Fibulin-3 was initially found to exert antiangiogenic effect [45], but in recent years, some studies had reported that fibulin-3 could promote angiogenesis, especially in pancreatic adenocarcinoma and cervical cancer, they found that fibulin-3 gene transfection elevated VEGF expression and microvessel density [17,18]. Since fibulin-4 is highly homologous to fibulin-3 and fibulin-5, we speculate that fibulin-4 may play a significant role in tumor angiogenesis. Pearson correlation tests of MVD and VEGF expression versus the corresponding expression of fibulin-4 revealed strong direct correlations. At the same time, as with fibulin-4, VEGF was also highly expressed in highly invasive subclones. These results partly validated our speculation that fibulin-4 might promote cervical tumor angiogenesis. Of course, further studies are needed to confirm our speculation, such as vascular formation test, nude mice test, RNAi experiment, etc.

CA125 is one of the most important biomarkers for ovarian cancer. It is often used for monitoring treatment effect and detecting recurrence in ovarian cancer. Elevated levels of CA125 have also been found in benign conditions such as endometriosis, pregnancy, ovulatory cycles, liver diseases, congestive heart failure, and infectious disease such as tuberculosis. CA125 alone is not a useful diagnostic marker for ovarian cancer [46,47]. CA19-9 is initially recognized as a marker for human colon cancer and pancreatic cancer [48,49]. Reports have showed that CA19-9 is also significantly elevated in patients with ovarian cancer, especially in mucinous cystadenocarcinoma [50]. In our research, high serum levels of fibulin-4, CA-125 and CA19-9 were all found in ovarian carcinoma when compared with healthy control and benign ovarian tumor, and high fibulin-4, CA-125 and CA19-9 levels were associated with low differentiation, advanced stage and positive lymph node status in ovarian carcinomas. Fibulin-4 combined with CA-125 and CA19-9 lead to a superior diagnostic specificity, positive predictive value and positive likelihood ratio. In recent years, fibulins have been recognized as biomarkers for many diseases, such as osteoarthritis, pleural mesothelioma and breast carcinoma. Fibulin-3 and fibulin-4 may play pathogenic roles in osteoarthritis [51,33]. The plasma fibulin-3 and fibulin-1 levels were elevated in patients with mesothelioma and breast carcinoma, respectively [52,53]. Newer specific biomarkers can help detect diseases at an earlier stage and tailor treatment strategies for individualized management. Combined with CA-125 and CA19-9, fibulin-4 may be advantageous to the early detection of ovarian carcinoma.

## Conclusion

Fibulin-4 is a newly identified gene that is overexpressed in ovarian cancer and associated with poor prognosis. Combined with CA-125 and CA19-9, serum levels of fibulin-4 may be helpful to early diagnosis and prognosis judgment. Fibulin-4 may possibly also serve as a novel therapeutic target in patients with ovarian cancer in the future.
